# Nuclear ERK: Mechanism of Translocation, Substrates, and Role in Cancer

**DOI:** 10.3390/ijms20051194

**Published:** 2019-03-08

**Authors:** Galia Maik-Rachline, Avital Hacohen-Lev-Ran, Rony Seger

**Affiliations:** Department of Biological Regulation, Weizmann Institute of Science, Rehovot 7610001, Israel; galia.maik-rachline@weizmann.ac.il (G.M.-R.); avital.levran@weizmann.ac.il (A.H.-L.-R.)

**Keywords:** mitogen-activated protein kinase (MAPK), Beta-like importins, nuclear translocation, nuclear substrates, negative feedback loop

## Abstract

The extracellular signal-regulated kinases 1/2 (ERK) are central signaling components that regulate stimulated cellular processes such as proliferation and differentiation. When dysregulated, these kinases participate in the induction and maintenance of various pathologies, primarily cancer. While ERK is localized in the cytoplasm of resting cells, many of its substrates are nuclear, and indeed, extracellular stimulation induces a rapid and robust nuclear translocation of ERK. Similarly to other signaling components that shuttle to the nucleus upon stimulation, ERK does not use the canonical importinα/β mechanism of nuclear translocation. Rather, it has its own unique nuclear translocation signal (NTS) that interacts with importin7 to allow stimulated shuttling via the nuclear pores. Prevention of the nuclear translocation inhibits proliferation of B-Raf- and N/K-Ras-transformed cancers. This effect is distinct from the one achieved by catalytic Raf and MEK inhibitors used clinically, as cells treated with the translocation inhibitors develop resistance much more slowly. In this review, we describe the mechanism of ERK translocation, present all its nuclear substrates, discuss its role in cancer and compare its translocation to the translocation of other signaling components. We also present proof of principle data for the use of nuclear ERK translocation as an anti-cancer target. It is likely that the prevention of nuclear ERK translocation will eventually serve as a way to combat Ras and Raf transformed cancers with less side-effects than the currently used drugs.

## 1. Introduction

Extracellular signal-regulated kinases 1/2 (ERK) belong to the family of mitogen-activated protein kinases (MAPK) that operate within signaling cascades that transmit extracellular signals to their intracellular targets. As such, the MAPK cascades are central signaling components that regulate fundamental cellular processes including proliferation, differentiation, and stress response [1,2,3]. These cascades transmit signals by a sequential activation of protein kinases organized in 3–5 tiers termed MAP4K, MAP3K, MAPKK, MAPK, and MAPKAPK. The three central tiers are considered as the essential core unit, while the other two appear in some of the cascades and may vary among cells and stimuli. Four MAPK cascades have been elucidated thus far, termed according to the component of the MAPK tier. These are: Extracellular Signal-Regulated Kinase (ERK) 1/2, c-Jun N-terminal Kinase (JNK), p38MAPK, and ERK5. In this review, we focus on ERK [4,5,6], whose cascade is composed of several kinases at the MAP3Ks tier (mainly Rafs, but also MOS, TPL2, MEKK1, and MLTKα/β), MEK1/2 at the MAPKK tier, ERK1/2 at the MAPK tier, and several MAPKAPKs at the next tier (RSK, MNK, MSK, MK3, and MK5).

Being responsible for various fundamental cellular processes, the ERK cascade is tightly regulated. Among such regulators are dual specificity phosphatases [7,8,9,10], scaffold proteins [11,12,13,14], duration and intensity of the signals [15], and dynamic subcellular localization that compartmentalize the components of the cascade [5,16,17]. The central importance of the ERK cascade indicates that dysregulation of ERK would be detrimental to the cells, and ultimately to the organism. Indeed, hyperactivation of the various components was shown to induce several diseases, including cancer, inflammation, and developmental and neurological disorders [1,18,19,20,21,22,23,24,25]. Since ERK1 and ERK2 are very similar to each other, we will continue to use the term ERK in its singular form, even though it refers to each one of the two isoforms. In this review, we focus primarily on nuclear ERK. More specifically, we discuss the nuclear translocation of ERK, how this process regulates ERK function, and its effect on nuclear substrates. In addition, we review the role of ERK in cancer, emphasize the role of nuclear ERK in this disease, and show that prevention of the nuclear translocation of ERK serves as a therapeutic tool. Finally, we also compare the nuclear translocation of ERK to that of other signaling molecules, and discuss how this translocation is dysregulated in cancer, and how this dysregulated translocation can provide a novel target to combat cancer.

## 2. The Role of ERK Cascade in Cancer and as a Therapeutic Target

Dysregulation of the RAS-ERK pathway is a major trigger in the development of most cancer types. Hyperactivation of the ERK cascade is seen in most cancers, and activating mutation of the pathway are the most abundant oncogenes in all cancers. The different components of the cascade are highly mutated in human cancer. Driver mutation of RAS (mainly K-Ras) is the most frequently mutated oncogene as it appears in ~30% of all cancer types [26], or in ~10% of all cancer patients [27]. Mutations in RAFs (particularly B-Raf) have been found in ~8% of all cancers [28] as well as in all cancer patients [27]. MEK mutations are less frequent (~1%), while almost no primary disease-driving mutations of ERK have been reported thus far [27]. The relatively large number of mutations, and the fact that ERK is activated indirectly even by oncogenes that are not upstream of ERK, has led to major efforts towards the development of different inhibitors targeting the various components of the RAS-ERK pathway. Some of these efforts achieved encouraging results, leading to an upsurge in oncology therapy.

Combinations of the approved RAF and MEK inhibitors [29] are routinely used in the clinic and benefit mainly melanoma patients. Recently, ERK inhibitors were also developed, and these drugs are now in different stages of clinical trials (SCH772984 [30], MK-8353 [31], and others [32,33]). However, the main drawback with all these inhibitors is that their effect is limited. Despite the fact that ERK is hyperactive in many cancers, the inhibitors are effective in only a few specific cancer types. More problematic is the fact that even in those sensitive cancers, a resistance to the drugs is developed after a relatively short period of successful treatment [19,34,35]. In most cases, the resistance is induced by reactivation of ERK, while in other cases, it is caused by hyperactivation of alternative survival-related pathways (PI3K, Wnt, etc. [35]). Several molecular mechanisms have been implicated in the development of resistance, and those are extensively described elsewhere [35]. Of note for this chapter is the fact that the inhibitors block ERK activity and therefore also the negative feedback loops that it initiates, causing hyperactivation of upstream signaling. Therefore, designing inhibitors that do not interfere with the negative feedback loops should be of great value. In summary, effective targeting of the RAS-ERK pathway is still of great interest, and blocking the ERK cascade is considered a prime target for the treatment of many cancers that are resistant to the RAF and MEK inhibitors. As a consequence, a large number of next-generation RAF and MEK inhibitors, along with new ERK-specific inhibitors are under investigation. Our recent efforts in using the process of nuclear ERK translocation as a proof of concept for the development of drugs that do not block the negative feedback loop of the pathway and therefore cause less resistance are described below.

## 3. Translocation of ERK to the Nucleus

Compartmentalization and dynamic changes in cellular localization are important mechanisms that regulate ERK signaling specificity. It was shown that ERK directly phosphorylates hundreds of substrates that are localized either in the cytoplasm, in various organelles, or in the nucleus [36,37]. Indeed, ERK-mediated phosphorylation of substrates in these compartments changes the outcome of the signals [38]. Importantly, it was shown that proliferation is mediated mainly by ERK activity in the nucleus, while other activities such as differentiation are correlated better to ERK activity in the cytoplasm [39,40]. Since ERK is localized in the cytoplasm of resting cells, it is clear that it is the nuclear translocation [41] that is required to activate ERK’s nuclear substrates and regulate the relevant cellular processes [5,42]. Our group has extensively studied the nuclear translocation of MAPKs to the nucleus, focusing initially on the mechanisms that mediate nuclear translocation of ERK upon stimulation. Since ERK does not contain the classical nuclear localization signal (NLS), we revealed that stimulation-induced translocation of ERK does not occur through the canonical NLS and (Imp) α/β nuclear shuttling machinery [43]. Instead, we showed that in resting state, ERK is localized in the cytoplasm due to its interaction with anchoring proteins [12,44].

Upon stimulation, ERK is activated by phosphorylation of its regulatory Tyr and Thr [45] that also induce a conformational change that releases it from the anchoring proteins [46]. This exposes additional Ser residues (SPS motif) within a unique nuclear translocation signal (NTS) located within the kinase insert domain to undergo phosphorylation [47] mainly by protein kinase CKII [48]. Unlike previous interpretations [49], our crystallographic studies [48] suggest that the SPS motif is fully exposed to allow its phosphorylation, and is even more exposed after phosphorylation. The phosphorylation of ERK’s SPS motif facilitates binding to importin7 (Imp7), which then escorts ERK to the nucleus via the nuclear pores. In the nucleus, the small GTPase Ran dissociates ERK from Imp7, leading to an accumulation of the kinase in the nucleus and the export of Imp7 back to the cytoplasm [50]. Interestingly, we have found that this mechanism is specific for ERK and a few other proteins [47,51,52]. It is distinct from the mechanisms used by other signaling proteins [51,53], and unlike previous assumptions [49], it accounts for the translocation of most, if not all, stimulated ERK molecules [54].

## 4. Nuclear Functions of ERK

The rapid and robust translocation of ERK into the nucleus, where it induces proliferation and other processes, was the impetus for revealing the underlying mechanisms which mediate ERK’s nuclear activities. In order to better understand this process, we followed the subcellular localization of ERK substrates that have been identified thus far [37]. Substrates localization was determined using data from the UniProt and the Human Protein Atlas. Out of more than 600 confirmed substrates, we have identified ~125 proteins that are solely nuclear (Table 1). These substrates include a large number of transcription factors such as chromatin modifying enzyme, nuclear envelope as well as structural proteins and others, which are all dependent on ERK nuclear translocation for their phosphorylation. We also identified 44 proteins that appear mainly in the nucleus, but also in other locations (Table 2). The reason for the distinct localizations of these proteins is not always clear, but may, in some cases, be due to stimulated translocation either in or out of the nucleus. In addition, the site in which these proteins are phosphorylated by ERK has not been fully studied, and is not necessarily dependent on ERK translocation. Many of these proteins are transcription factors or modifiers, and some of them shuttle in or out of the nucleus upon stimulation (e.g., SMADs [55]). It is likely that additional ERK substrates do exist, but those are not listed here.

Nuclear ERK seems to play an important role mainly in inducing and regulating stimulated proliferation or oncogenic transformation [39,54,56]. It does so by activating transcription factors and other regulatory components, mainly by their phosphorylation [57]. These activated proteins, in turn, induce the expression of proteins that further transmit the signals to initiate and regulate ERK-dependent processes. For this reason, we studied the changes in nuclear substrates in cancer. Using the expression databases TCGA Pan-Cancer Dataset, we found that the expression of 62 proteins is significantly changed (above 1.4-fold, Table 1 and Table 2), among them 22 proteins show increased expression and 40 proteins decreased expression. It is likely that the increased expression is linked to signals that initiate or maintain transformation, while the decreased expression is linked to downregulating components (e.g., p21Cip/CDKN1A [58]). Obviously, the phosphorylation that initiates the transcription of the genes encoding these proteins is distinct from the phosphorylation of the synthesized, mature proteins, which regulate their activity. Moreover, the phosphorylation of the expressed proteins by the constitutively active ERK is clearly important to maintain carcinogenesis, and their further study may lead to a better understanding of ERK-related processes in cancer. Below, we provide several examples for the mechanisms by which ERK phosphorylation affects carcinogenesis via cancer-induced nuclear proteins. The selected proteins represent mainly the biggest group of such proteins namely transcription factors (e.g., c-Myc, c-Fos). However, it is important to mention that not all nuclear ERK effectors belong to this group, and as a representative of the other groups we describe transcriptional suppressors and the protein kinase DYRK1B.

## 5. Nuclear Substrates of ERK in Cancer Development and Maintenance

One of the main cancer-associated ERK substrates is c-Myc [59,60], which is a transcription factor for cell cycle progression, and other fundamental cellular processes [61,62]. The main way by which c-Myc becomes an oncogene is its elevated expression, which occurs by several mechanisms such as retroviral transduction, or gross genetic abnormalities that affect its gene. In addition, c-Myc can be deregulated by cancer-related signaling, including phosphorylation by ERK [60], alterations in c-Myc mRNA, and protein stabilization [63]. Phosphorylation of c-Myc by ERK on Ser62, usually due to RAS activation, is one of the most important mechanisms that keeps the overexpressed c-Myc stabilized and is responsible for its accumulation in various cancers [59,64]. Dephosphorylation of Ser62 of c-Myc by protein phosphatase 2A (PP2A) causes ubiquitination of c-Myc and targets it for degradation [65]. Indeed, mutation of the ERK phosphorylation site that stabilizes c-Myc expression serves as an oncogene in some cancers as well [66]. Importantly, we have shown that nuclear translocation of ERK is indeed essential for the nuclear phosphorylation of c-Myc, which might serve as one of the main ways by which ERK induces cancers [54].

Another target of ERK that plays an important role in carcinogenesis is c-Fos, which is a transcription factor that is a vital component in the induction of proliferation [67]. The induction of c-Fos shortly after mitogenic stimulation is mainly mediated by the ERK cascade via the transcription factor Elk1. Elk1 is expressed in resting cells, and is one of the first transcription factors that is phosphorylated and activated by ERK [68]. After its phosphorylation, Elk1 is engaged in the transcriptional complex that binds to the promotor of c-Fos, and induces the expression of the latter. When the c-Fos protein is expressed, it can be phosphorylated by ERK in the nucleus on Ser374, and this phosphorylation enhances the stability and transforming activity of c-Fos [69,70,71]. Importantly, the accumulation of c-Fos is highly dependent on the duration of ERK activity, and is used as a readout mechanism of the cell fate-determining signaling of ERK [72]. When ERK activation is transient, its activity declines before c-Fos is expressed, and therefore, stabilizating c-Fos phosphorylation and stabilization does not occur, resulting in its limited transcriptional activity. However, when ERK is activated for a longer duration (more than 30 min), the phosphorylation of c-Fos does occur, and this causes c-Fos stabilization and modifies its transcriptional activity. Obviously, ERK activity in cancer is elevated, and therefore, c-Fos is always phosphorylated and stabilized, allowing for its cancer-initiating processes to occur [73]. We have shown that Elk1 and c-Fos phosphorylation, as well as c-Fos expression, are dependent on nuclear ERK translocation [54]. Recently, it was also shown that the protein MTBP interrupts the ERK-Imp7 binding [74]. As a consequence, ERK does not translocate to the nucleus, and this results in reduced Elk1 phosphorylation as well as reduced expression of Elk/c-Fos target genes.

Although c-Myc and c-Fos are bona-fide oncogenic transcription factors, other nuclear transcription factors and suppressors mediate ERK activity in cancer as well. One example is the transcription factor high mobility group-box factor (UBF), which is phosphorylated by ERK on its HMG-box, leading to its activation [75,76]. The rRNA transcription activity of UBF can accelerate cell cycle, and the hyperphosphorylation of UBF may be involved in carcinogenesis [77]. Another way of transmitting ERK-dependent nuclear activity is by inhibition of tumor suppressor proteins, and example for such mechanism is Tob [78]. This transcriptional suppressor demonstrates antiproliferative function by binding to transcription factors in the nucleus and suppressing the expression of cyclin D1 and other regulatory genes [79,80]. Importantly, ERK phosphorylates Tob in the nucleus on Sers 152, 154, and 164 and these phosphorylations inhibit Tob activation and may support carcinogenesis [81]. Another example is Foxo3a, a tumor suppressor transcription factor, which is phosphorylated by active nuclear ERK on Sers 294, 344, and 425 [82,83]. These phosphorylations support the interaction of the Foxo3a with MDM2 that adds ubiquitin and targets Foxo3a for degradation. In contrast, it was shown that a non-phosphorylated Foxo3a mutant displays a strong inhibition of cell proliferation and tumorigenicity [82,83]. The phosphorylation probably occurs in the nucleus, although in similarity to Foxo1 [84], Foxo3a may be exported to the cytoplasm after its phosphorylation, which together with the degradation of the protein lead to its suppression [85]. Finally, an example of ERK target other than transcription factor is the protein Ser/Thr kinase DYRK1B, which plays a role in cell cycle progression and survival [86], as well as in carcinogenesis [87]. Importantly, its phosphorylation by ERK on Ser421 activates it, and can participate in cancer induction downstream of ERK [88]. Thus, nuclear ERK localization is essential for cancer formation by enhancing oncogenic signals or inhibiting tumor suppressors in many cancer types.

## 6. Nuclear Translocation of Other Signaling Proteins

Although we have extensively described the nuclear translocation of ERK, many other signaling proteins translocate to the nucleus in response to various stimuli in order to exert their function. For example, it is apparent that mitogens induce a rapid and robust translocation of at least 50 distinct proteins, including: RSK1-4 [41], MEK1/2 [89], several isoforms of PKC [90,91,92,93,94], EGFR [95], JNK1/2 and p38α−β [53], and EGR1 [96]. Although many of these proteins are important for the regulation of proliferation and carcinogenesis, their mechanism of translocation is not fully elucidated. Yet, it was reported that some of the proteins have the canonical NLS, which is differentially exposed upon stimulation and likely interacts with Impα/β (e.g., ERK5 [97]). In addition, other proteins were shown to use specific NLSs/NTSs-mediated binding to members of the β-like importin family for their translocation [47,53,96,98]. Our working hypothesis is that this group of importins is particularly important for stimulation-induced translocation, while the canonical mechanism of translocation is mainly involved in the house-keeping, non-stimulated translocation of nuclear proteins. Therefore, it is apparent that there are at least three mechanisms of stimulated nuclear translocation with distinct regulations. The NLS-mediated mechanism typified below by ERK5, the β-like importins-dependent mechanism which is represented below by p38MAPK, and unrelated mechanisms as shown below for β-catenin. These stimulated nuclear translocations have been described in cancer cells. More studies are required to fully reveal the underlying mechanisms in order to potentially use them as targets for combating cancer.

ERK5—A signaling protein that is highly regulated by nuclear translocation is ERK5 (big-MAPK; BMK1). This 105 kDa protein, which belongs to the MAPK family, is ubiquitously expressed and is activated by its only upstream kinase, MEK5, in response to growth factors and stress stimulations. The ERK5 cascade has been associated mainly with stress as well as proliferation [99]. These effects are mediated by nuclear transcription factors such as MEF2C, AP-1, and c-Fos [100], or by ERK5 that can act as a transcription factor by itself [101]. Therefore, translocation/localization of ERK5 to the nucleus is essential in regulating ERK5-mediated gene transcription, and cellular processes. The ERK5 subcellular localization is dynamic and may vary depending on the cell type and its stimulated conditions [102,103,104,105]. In some cells, ERK5 and MEK5 are localized in the nucleus, and are activated by a translocating MEKK2 [102]. However, in other cells, it is kept in the cytoplasm by its exposed nuclear export signal (NES, [106]), and by binding to anchoring proteins, such as Hsp90 and Cdc37 dimer [107]. Upon stimulation, ERK5 detaches from the anchoring proteins, and undergoes a conformational change to hinder its NES and expose its hidden NLS motif that allows its nuclear translocation [106]. In the nucleus, ERK5 activates transcription to induce a set of processes that allow its involvement in proliferation, stress response, and cancer [100,108,109,110]. Thus, inhibitors of ERK5 activity or silencing the protein are antiproliferative and block tumor growth in animal models [100,111], and efforts are being taken to develop more efficient inhibitors for clinical use.

P38MAPK—P38 is a group of four protein kinases α−δ, which are very similar to each-other [2,112]. They are activated mainly by MKK3 or MKK6 and to some extent by MKK4, all of which are able to induce full activation of the different p38 isoforms. As a group member of the MAPK family, p38 participates in the induction and regulation of a large number of cellular processes and is mainly influential in stress responses. Its dysregulation is involved primarily in inflammation [113], autoimmune diseases [114], and also cancer [115]. As other MAPKs, p38 nuclear localization is essential for mediating the full effects of this kinase. Indeed, similarly to ERK5, its subcellular localization is dynamic and may vary depending on the cell type and its stimulated conditions. In some resting cells, p38 is localized in the nucleus and is exported after stimulation [116,117], while in many others it is cytoplasmic and translocates to the nucleus upon stimulation [53,118]. The mechanism of this translocation involves a stimulated binding to a dimer of either Imp7/3 or Imp9/3 that escorts the p38 to the nucleus [53]. Other mechanisms of stimulated translocation might exist as well [49]. We have recently reported that inhibition of this translocation reduces proliferation of triple negative breast cancer and other cancers, reduces inflammation in a colitis model, and significantly blocks colitis-induced colon cancer in an animal model [98]. This indicates that β-like importins-mediated p38 nuclear translocation plays a significant role in several types of cancers, which can be targeted by p38 specific translocation inhibitors.

β-catenin—The β-catenin protein is a critical signaling molecule of the Wnt signaling pathway that plays a role primarily in proliferation and development [119,120,121]. The binding of Wnt to its receptor Frizzled leads to inhibition of a destruction complex composed of APC and Axin. This facilitates the blockage of β-catenin phosphorylation, and therefore, its stabilization and nuclear accumulation. There, β-catenin binds TCF/LEF-1 transcription factors to transactivate genes involved in differentiation, proliferation, and cancer [122]. Indeed, mutations in the components of the Wnt pathway that induce nuclear β-catenin accumulation play a role in several cancers, mainly CRC [27]. Moreover, β-catenin in the nucleus correlates with elevated clinical tumor grade, and nuclear staining of β-catenin was shown in the invasive front of tumors [122]. Regarding its mechanism of nuclear translocation, it was initially thought that β-catenin translocates by direct contact with the nuclear pore complex [120,123]. However, later studies showed that, at least in part, nuclear β-catenin translocation is dependent on SMAD3 [124], or may involve RAPGEF5 [125]. These findings indicate that the pathway does not operate via a diffusion-mediated shuttling, but rather, that the NLS/NTS of β-catenin has not been identified yet. This process of nuclear translocation is well-regulated by signaling pathways including that of JNK [126]. However, more information is needed to understand the regulation of nuclear β-catenin translocation and its potential use for combating cancer.

## 7. Targeting ERK Nuclear Translocation for Cancer Treatment

As mentioned earlier, the RAS-ERK pathway is highly mutated in cancer, and is considered a prime target in terms of development of inhibitors. A few of these inhibitors are already in therapeutic use, but all of them are problematic as they are effective only in limited cancer types and their usage results in the development of resistance 2–18 months after administration. Since this pathway is a prime target for combating cancer, inhibition of other steps of the cascade should be considered in order to overcome the development of resistance. For this purpose, we targeted ERK nuclear translocation as a means of inhibiting its proliferating outcome, while retaining ERK’s cytosolic functions. This lack of cytoplasmic inhibition should have less side effects, and most importantly, will not inhibit negative feedback loops that are strongly affected by complete inhibition of ERK activity [19,35,127]. Since prevention of the negative feedback loops is one of the main mediators of drug resistance, such inhibitors of nuclear translocation are predicted to dramatically reduce the development of resistance.

**Figure 1:**
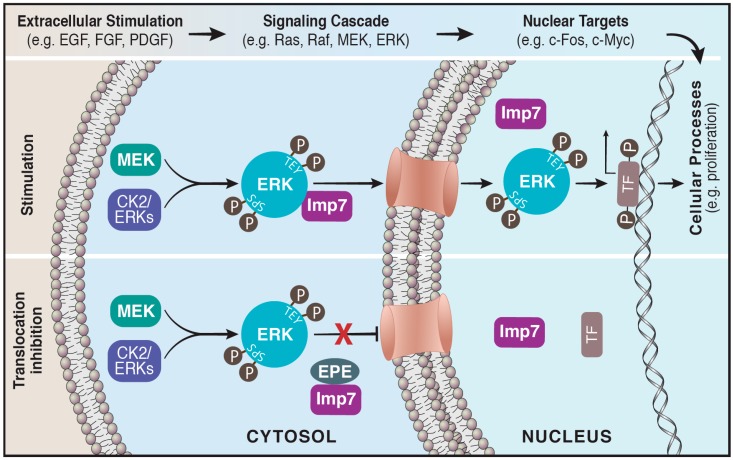
The nuclear translocation of ERK is blocked by the EPE peptide. Active ERK translocates to the nucleus via its interaction with importin7. Cytosolic ERK is activated initially by phosphorylation on its regulatory Tyr and Thr residues (TEY) followed by phosphorylation on its Ser residues (SPS) located within the nuclear translocation sequence (NTS). This facilitates the binding of the beta-like importin, Imp7, to ERK escorting it to the nucleus via the nuclear pores, where it modulates a large number of targets such as transcription factors (**Upper panel**). A myristoylated NTS-derived phosphomimetic peptide (EPE peptide) specifically blocks the interaction of Imp7 with ERK thereby inhibits nuclear translocation of ERK (**Lower panel**).

Taking into consideration that the nuclear translocation of ERK is specific for this MAPK component, we designed a synthetic myristoylated peptide (EPE peptide, [54]), which specifically blocked ERK interaction with Imp7 and thereby nuclear translocation (Figure 1). In culture, the peptide specifically induced apoptosis of mutated BRAF melanoma cells, inhibited proliferation of RAS transformed as well as other cancer cells, but had no effect on immortalized cells [54]. This important result may hint at the therapeutic potential of the EPE peptide as it affects only the cancerous cells but not the surrounding milieu. Moreover, the peptide even inhibited the growth of PLX4032 (RAF inhibitor)- and U0126 (MEK inhibitor)-resistant melanoma cells. In a later study, we demonstrated that the EPE peptide also inhibited proliferation of several NRAS and NF1 mutant melanomas [128]. Importantly, combining the EPE peptide and the MEK inhibitor trametinib showed synergy in inhibiting proliferation of some NRAS mutant melanomas resistant to each drug alone, due to the partial preservation of negative feedback loops.

Moreover, the peptide was shown to be highly effective in xenograft models, as it inhibited the growth of breast cancer, colon cancer, and melanoma, and completely prevented the growth of BRAF mutated melanoma. Importantly, as excepted from the retained activity of the feedback loops, the peptide was much more effective than PLX4032 in preventing tumor recurrence in melanoma xenografts. There was no recurrence of melanoma in any of the EPE treated mice, whereas 60% of the PLX4032 treated mice exhibited relapse and tumor regrowth [54]. Therefore, our results support the concept of targeting nuclear transport in order to develop anticancer agents. More specifically, inhibition of ERK nuclear translocation is a novel therapeutic approach to combat various RAS, RAF, or ERK related cancers. This approach may be beneficial by affecting mainly nuclear ERK activity in relevant cancers, while retaining cytoplasmic ERK-induced negative feedback loops, therefore reducing resistance mechanisms.

## 8. Summary

The response of cells to mitogenic or oncogenic stimulation results in the activation of several signaling pathways including primarily that of ERK. One of the hallmarks of these stimulations is the rapid and robust nuclear translocation of the MAPK components of each of the pathways. This is essential for the activation of transcription factors and chromatin modifiers, and consequently for regulated cellular processes. Although it is clear that the nuclear translocation is essential for important processes such as proliferation and stress response, much less is known about the mechanism of this translocation, as many of the proteins do not contain NLS. We have found that ERK translocation is mediated by β-like importins such as Imp7. It is possible that these and other β-like importins participate in the shuttling of other signaling components as well.

Since nuclear translocation is a central regulator of the ERK signaling cascade, dysregulation of translocation and accumulation of active ERK or other signaling components in the nucleus plays a role in pathologies such as carcinogenesis. Kinase inhibitors of this pathway have had an initially good response, but suffer mainly from the development of resistance. To overcome this problem, we targeted ERK nuclear translocation as a means of inhibiting nuclear ERK activity without affecting ERK cytosolic functions. This inhibition resulted in an eradication of tumor growth of serval RAS- and RAF-transformed cancers, without the development of resistance over prolonged treatment. Thus, the nuclear translocation of ERK, and probably of other signaling pathways, can serve as a good target for cancer, and should be further developed for clinical use.

## Figures and Tables

**Table 1:** Cancer-induced changes in gene expression of solely nuclear ERK substrates. Listed are direct ERK substrates (taken from http://sys-bio.net/erk_targets/targets_all.html) that are constantly localized in the nucleus (as per data from UniProt and the Human Protein Atlas). Gene expression of these proteins is presented in log-transformed normalized counts of tumor vs. normal tissues (taken from TCGA Pan-Cancer Dataset), and compared by *t*-tests. Significance is presented by *p*-value. Fold change presents the ratio between the gene expression in tumor vs. normal tissues in regular and not logarithmic scale. Negative values refer to downregulated genes and positive values refer to upregulated genes in tumors. Proteins are assigned in the table from highest to lowest in downregulated genes and lowest to highest in upregulated genes. * The nuclear proteins UBF, NSMF, RBFOX2, and SRSF11 do not exist in TCGA Pan-Cancer Dataset, therefore we did not refer to their expression in tumor and normal tissues.

Gene	Gene Expression		*p*-Value	Fold Change
	Tumor	Normal		
*MYOCD*	3.53	6.16	1.4 × 10^−119^	−6.19
*NR0B2*	2.20	4.39	9.1 × 10^−43^	−4.55
*PGR*	5.45	7.59	3.6 × 10^−94^	−4.43
*FOS*	11.72	13.84	3.2 × 10^−172^	−4.34
*EGR1*	11.63	13.67	2.2 × 10^−16^	−4.11
*DUSP1*	11.58	13.56	2.2 × 10^−230^	−3.95
*THRB*	7.62	9.40	2.9 × 10^−214^	−3.44
*IRX2*	5.20	6.81	1.3 × 10^−31^	−3.06
*PPARG*	7.39	8.96	5.0 × 10^−109^	−2.96
*TAL1*	5.28	6.74	2.7 × 10^−133^	−2.76
*NR4A2*	7.74	9.19	3.5 × 10^−94^	−2.72
*ESR1*	6.84	8.16	3.6 × 10^−41^	−2.50
*MITF*	7.83	9.05	2.8 × 10^−93^	−2.33
*ARRB1*	8.25	9.32	3.0 × 10^−92^	−2.10
*RORA*	8.31	9.38	1.8 × 10^−132^	−2.09
*JUN*	12.03	13.09	5.0 × 10^−106^	−2.08
*RPS6KA5*	6.83	7.65	8.3 × 10^−69^	−1.77
*JUNB*	11.68	12.47	7.1 × 10^−53^	−1.73
*NCOA2*	8.61	9.37	4.9 × 10^−60^	−1.70
*TOB1*	10.46	11.11	5.3 × 10^−77^	−1.56
*ETV3*	7.01	7.61	8.2 × 10^−45^	−1.53
*ETS1*	10.65	11.23	1.8 × 10^−44^	−1.50
*GATA1*	0.88	1.46	1.7 × 10^−31^	−1.49
*NFATC1*	8.07	8.62	1.9 × 10^−44^	−1.47
*PDCD4*	11.09	11.60	7.2 × 10^−32^	−1.43
*RXRA*	10.90	11.41	3.3 × 10^−72^	−1.43
*MKL2*	9.86	10.27	1.9 × 10^−33^	−1.34
*CREM*	8.64	9.04	2.0 × 10^−30^	−1.32
*ATF2*	8.49	8.85	1.0 × 10^−39^	−1.29
*DCP1A*	9.20	9.56	3.5 × 10^−65^	−1.28
*SNAI2*	7.85	8.19	9.8 × 10^−12^	−1.27
*ETV1*	8.04	8.37	2.0 × 10^−8^	−1.26
*CEBPA*	9.19	9.50	1.2 × 10^−5^	−1.24
*CDKN1A*	11.39	11.69	5.3 × 10^−10^	−1.24
*ESR2*	3.71	3.98	1.8 × 10^−7^	−1.20
*EP300*	10.94	11.19	2.7 × 10^−27^	−1.19
*CIITA*	7.74	7.95	5.8 × 10^−5^	−1.16
*PHF2*	10.10	10.30	1.0 × 10^−19^	−1.15
*HNRNPH2*	11.13	11.30	2.1 × 10^−50^	−1.13
*SSBP3*	9.46	9.61	2.5 × 10^−11^	−1.11
*FBXW7*	8.59	8.73	3.8 × 10^−7^	−1.10
*HDAC6*	10.22	10.34	8.7 × 10^−6^	−1.09
*RB1*	10.07	10.19	5.1 × 10^−8^	−1.08
*NUP98*	11.03	11.14	2.0 × 10^−14^	−1.08
*ALOX5*	8.31	8.42	0.15	−1.08
*GTF2I*	11.10	11.20	1.1 × 10^−3^	−1.07
*THRAP3*	11.58	11.67	1.1 × 10^−13^	−1.07
*SP1*	11.25	11.35	1.0 × 10^−6^	−1.07
*POLR2A*	12.20	12.29	2.4 × 10^−5^	−1.07
*LMNA*	12.92	13.00	0.02	−1.06
*CDKN1B*	10.46	10.54	2.0 × 10^−5^	−1.06
*MYC*	10.60	10.67	0.23	−1.05
*GLI2*	6.09	6.15	0.33	−1.04
*TNKS1BP1*	11.86	11.90	0.08	−1.03
*NUMA1*	12.89	12.93	0.07	−1.03
*NUP214*	10.49	10.53	0.01	−1.03
*NUP153*	10.21	10.24	0.08	−1.02
*ELK4*	7.07	7.10	0.43	−1.02
*H3F3B*	13.57	13.59	0.48	−1.01
*SP3*	10.69	10.70	0.45	−1.01
*CEBPB*	10.07	10.08	0.81	−1.01
*HMG20A*	9.64	9.65	0.46	−1.01
*AEBP1*	11.97	11.95	0.76	1.01
*SRRM2*	13.32	13.30	0.46	1.01
*NEUROD1*	0.99	0.96	0.68	1.02
*MAFA*	0.68	0.65	0.44	1.02
*TERF2*	9.25	9.22	3.5 × 10^−3^	1.02
*HIF1A*	11.66	11.62	0.17	1.03
*MED1*	10.17	10.12	0.02	1.03
*MKNK1*	9.01	8.96	0.02	1.03
*RPS6KB1*	9.48	9.41	6.2 × 10^−5^	1.05
*XRN2*	10.91	10.83	3.2 × 10^−8^	1.06
*PAPOLA*	11.77	11.69	4.2 × 10^−13^	1.06
*RRN3*	10.17	10.08	4.2 × 10^−9^	1.06
*HNRNPK*	13.51	13.39	2.3 × 10^−41^	1.09
*MAPKAPK2*	11.56	11.43	8.2 × 10^−12^	1.10
*SAFB2*	10.57	10.43	2.4 × 10^−11^	1.10
*NUP50*	10.26	10.12	1.5 × 10^−15^	1.10
*TPR*	11.44	11.29	1.2 × 10^−13^	1.11
*UBTF*	11.53	11.37	2.6 × 10^−25^	1.12
*MZF1*	8.35	8.18	9.1 × 10^−10^	1.13
*TP53BP1*	9.98	9.79	9.4 × 10^−10^	1.14
*KHDRBS1*	11.81	11.61	2.6 × 10^−48^	1.15
*PHOX2A*	0.83	0.63	1.7 × 10^−4^	1.15
*SF3B2*	12.30	12.08	1.2 × 10^−70^	1.16
*DYRK1B*	9.02	8.79	2.7 × 10^−17^	1.17
*TGIF1*	10.36	10.13	1.9 × 10^−16^	1.17
*EWSR1*	12.12	11.88	1.9 × 10^−68^	1.18
*HIST1H3A*	0.73	0.49	2.5 × 10^−20^	1.18
*DDX47*	10.13	9.88	2.5 × 10^−82^	1.19
*NCOA6*	10.61	10.36	2.3 × 10^−37^	1.20
*NCOR2*	11.93	11.66	4.3 × 10^−22^	1.21
*HNRNPD*	11.96	11.68	1.9 × 10^−74^	1.22
*BAZ1B*	11.14	10.85	1.7 × 10^−61^	1.22
*ERF*	10.21	9.91	6.5 × 10^−29^	1.23
*FAM103A1*	8.52	8.22	1.9 × 10^−66^	1.23
*REXO1*	9.57	9.27	3.9 × 10^−44^	1.24
*SUPT5H*	11.72	11.40	1.1 × 10^−105^	1.25
*CSNK2A1*	10.37	10.05	2.1 × 10^−89^	1.25
*RARG*	9.55	9.23	1.7 × 10^−10^	1.25
*TP53*	10.34	10.00	7.2 × 10^−37^	1.26
*SAFB*	10.96	10.62	5.7 × 10^−71^	1.27
*WIZ*	10.34	9.98	2.4 × 10^−80^	1.28
*ZC3HC1*	8.66	8.29	1.6 × 10^−102^	1.29
*PML*	10.91	10.54	4.4 × 10^−45^	1.29
*ELK1*	9.77	9.35	6.8 × 10^−109^	1.35
*GATA4*	2.77	2.25	6.3 × 10^−5^	1.44
*MYBBP1A*	10.22	9.56	1.4 × 10^−111^	1.57
*RUNX1*	10.24	9.56	4.8 × 10^−24^	1.61
*RUNX2*	7.40	6.70	1.9 × 10^−39^	1.62
*CCDC86*	9.51	8.81	3.6 × 10^−143^	1.62
*NR5A1*	1.28	0.47	4.6 × 10^−45^	1.74
*INCENP*	8.72	7.91	1.6 × 10^−84^	1.75
*TWIST1*	5.73	4.91	9.8 × 10^−19^	1.77
*CAD*	10.36	9.52	5.4 × 10^−150^	1.80
*PDX1*	2.03	1.14	5.2 × 10^−24^	1.84
*MAZ*	12.19	11.31	6.7 × 10^−173^	1.85
*TCF3*	10.71	9.77	5.2 × 10^−188^	1.92
*LMNB1*	9.62	7.90	3.2 × 10^−153^	3.28
*ESPL1*	7.68	5.59	4.7 × 10^−104^	4.25
*TOP2A*	10.37	7.10	6.8 × 10^−194^	9.64
*UBF	NA	NA	NA	NA
*NSMF	NA	NA	NA	NA
*RBFOX2	NA	NA	NA	NA
*SRSF11	NA	NA	NA	NA

**Table 2:** Cancer-induced changes in gene expression of nuclear ERK substrates that are localized in the nucleus and other organelles. Listed are direct ERK substrates localized in the nucleus as well as in at least one additional organelle. The data bases and calculations are similar to ones presented in Table 1. The additional localization of each of the substrates as appear in UniProt and the Human Protein Atlas is indicated as well. * The protein PRRC2A that appears in the nucleus, cytosol, and plasma membrane does not exist in TCGA Pan-Cancer Dataset, therefore we did not refer to its expression in tumor and normal tissues.

Gene	Gene Expression		*p*-Value	Fold Change	Other Localizations
	Tumor	Normal			
NR4A1	10.47	12.37	3.4 × 10^−118^	−3.73	cytosol
CBX7	9.50	10.94	1.9 × 10^−223^	−2.72	cytosol
CRYAA	0.99	2.24	4.1 × 10^−17^	−2.38	cytosol
ERG	7.80	8.98	1.4 × 10^−106^	−2.27	cytosol
RBPMS	9.75	10.88	1.3 × 10^−124^	−2.19	cytosol
FOXO1	9.25	10.36	2.7 × 10^−189^	−2.17	cytosol
CRY2	9.75	10.82	8.4 × 10^−219^	−2.09	cytosol
NR3C1	9.93	10.89	3.1 × 10^−161^	−1.95	cytosol + mitochondria
AIM1	9.64	10.57	2.4 × 10^−52^	−1.91	cytosol + microtubules
EGFR	9.23	10.06	2.5 × 10^−76^	−1.78	plasma membrane
ETS2	11.23	12.01	1.6 × 10^−69^	−1.72	cytosol + plasma membrane
NCOA1	10.21	10.74	3.5 × 10^−112^	−1.44	cytosol + plasma membrane
FGFR1	10.27	10.77	2.3 × 10^−24^	−1.42	plasma membrane
RPS6KA3	10.33	10.82	1.2 × 10^−85^	−1.40	cytosol
SMAD4	10.43	10.92	2.5 × 10^−158^	−1.40	cytosol
STAT5A	9.48	9.94	5.8 × 10^−36^	−1.38	cytosol
SORBS3	10.76	11.22	4.4 × 10^−72^	−1.37	focal adhesion sites
FOXO3	10.44	10.82	1.6 × 10^−48^	−1.30	cytosol
BCL6	9.96	10.29	1.4 × 10^−14^	−1.25	Golgi
CRY1	8.74	9.03	1.9 × 10^−33^	−1.22	microtubules
SREBF2	11.72	11.98	2.6 × 10^−24^	−1.20	mitochondria
WASL	10.75	11.00	4.9 × 10^−43^	−1.19	cytosol
SMAD3	10.45	10.70	7.8 × 10^−25^	−1.19	cytosol
SMAD2	10.63	10.79	3.6 × 10^−19^	−1.12	cytosol
SMAD1	9.25	9.40	9.9 × 10^−12^	−1.11	cytosol + plasma membrane
RPS6KA1	10.22	10.25	0.59	−1.02	cytosol
PIP5K1C	10.36	10.32	0.16	1.03	cytosol
MKL1	9.98	9.90	1.2 × 10^−4^	1.06	cytosol
RAI14	10.11	10.02	1.4 × 10^−2^	1.06	cytosol
NFATC4	8.76	8.59	1.4 × 10^−3^	1.12	cytosol
TGS1	9.07	8.91	3.1 × 10^−18^	1.12	cytosol
HNRNPH1	12.00	11.83	2.4 × 10^−21^	1.13	cytosol
ETV6	9.99	9.81	5.6 × 10^−9^	1.14	cytosol
STAT1	12.23	11.80	3.1 × 10^−43^	1.35	cytosol
PPP1R9B	10.73	10.29	5.6 × 10^−72^	1.36	cytoskeleton
DAZAP1	10.82	10.34	2.7 × 10^−113^	1.39	cytosol
POU5F1	3.91	3.42	1.5 × 10^−12^	1.40	cytosol
HSF1	10.98	10.48	3.3 × 10^−148^	1.41	cytosol
MYB	6.43	5.58	2.4 × 10^−11^	1.80	plasma membrane
SMC4	10.42	9.42	5.6 × 10^−157^	2.00	cytosol
MAGEA11	1.33	0.27	1.0 × 10^−159^	2.08	cytosol
PTTG1	8.68	6.19	1.2 × 10^−140^	5.63	cytosol
TPX2	9.75	6.59	5.7 × 10^−216^	8.95	microtubules
*PRRC2A	NA	NA	NA	NA	cytosol + plasma membrane
